# DeflVol: a model-derived volumetric descriptor of corneal deflection at highest concavity under Corvis ST air-puff loading

**DOI:** 10.3389/fbioe.2026.1933733

**Published:** 2026-10-19

**Authors:** Qi Ren, Zhaozhe Deng, Zhe Chu, Zeyu Pan, Pengxia Wan

**Affiliations:** 1 Department of Ophthalmology, First Affiliated Hospital, Sun Yat-sen University, Guangzhou, China; 2 School of Mechanical Engineering, Zhejiang University, Hangzhou, China; 3 Shandong Provincial Key Laboratory of Ophthalmology, Eye Institute of Shandong First Medical University, Qingdao, China

**Keywords:** corneal biomechanics, corneal deflection volume, Corvis ST, dynamic corneal response, Scheimpflug imaging

## Abstract

Air-puff-induced corneal deformation is inherently three-dimensional, motivating a volumetric descriptor that complements conventional displacement- and area-based metrics. We developed a closed-form, axisymmetric two-sphere model to estimate corneal deflection volume (DeflVol) at the highest concavity (HC) moment from the anterior corneal curvature radius, inverse curvature radius, and deflection amplitude. The model was evaluated using paired Pentacam AXL and Corvis ST measurements from 1,351 young emmetropic or myopic participants. Geometric consistency was assessed against Corvis ST outputs not used as model inputs; a geometry-based area-matching analysis and sensitivity analyses of corneal asphericity and regular anterior toricity were also performed. Predicted deflection area closely tracked measured area (*R* ^2^ = 0.99, slope = 0.96, mean bias = −0.181 mm^2^); the modeled overlap chord closely tracked peak distance (*R* ^2^ = 0.84, slope = 0.95, mean bias = +0.008 mm). DeflVol was 9.48 ± 1.72 μL and correlated strongly with deflection amplitude (*r* = 0.94, *p* < 0.001) and deflection area (*r* = 0.99, *p* < 0.001). Predicted area was 5.5% ± 1.4% lower than measured area; in the geometry-based area-matching sensitivity analysis, the model-implied DeflVol increase was 7.8% ± 2.2%, or approximately 0.7 μL Physiologically representative asphericity altered DeflVol by less than 1% on average, in 99% of eyes. DeflVol provides a computationally transparent, model-derived three-dimensional descriptor of corneal deformation that showed strong associations with established Corvis ST metrics.

## 1 Introduction

The dynamic corneal response (DCR) parameters obtained from Corvis ST have been widely used to characterize ocular resistance to deflection under air-puff loading (Vinciguerra et al., 2016a; Esporcatte et al., 2020). In particular, indentation- and displacement-related parameters provide clinically relevant information on biomechanical behavior and have contributed to the assessment of corneal properties in various ocular conditions (Roberts, 2014; Roberts et al., 2017; Vinciguerra et al., 2016b). However, because Corvis ST is based on high-speed Scheimpflug imaging, most currently available deflection descriptors are expressed as one-dimensional displacement amplitudes (e.g., deflection amplitude, DeflAmp) or two-dimensional area-based metrics (e.g., deflection area, DeflArea) (Esporcatte et al., 2020; Roberts et al., 2017). Although these parameters capture important aspects of corneal motion, they do not directly represent the overall geometric extent of deflection at the highest concavity (HC) moment.

Biomechanically, deflection induced by the Corvis ST air-puff is not a uniform displacement event; rather, it is a spatial deformation process that reflects both the cornea’s integrated structural response and the transient redistribution of intraocular contents under loading (Roberts, 2014; Xie et al., 2025; Joda et al., 2016). A parameter that describes deflection in volumetric terms may therefore complement conventional DCR metrics by providing a spatially integrated representation of deflection magnitude. Such an approach may be useful for characterizing corneal deformability under mechanical loading, a property potentially relevant to habitual stresses such as eye rubbing or forceful eyelid closure, and for comparing ocular biomechanical behavior across individuals. Constructing such a volumetric descriptor may therefore help assess individual susceptibility to biomechanics-related ocular disorders (Roberts and Dupps, 2014; Ma et al., 2021).

Related approaches have utilized finite-element and numerical simulation methods to connect measured corneal geometry with deflection behavior (Dorronsoro et al., 2012; Bao et al., 2018; Pang et al., 2024; Wang et al., 2022). These more detailed models provide valuable mechanistic insights but require computationally intensive iterative fitting. In contrast, a simplified geometric descriptor derived from clinically available measurements could offer a simpler, computationally transparent approach to characterizing the three-dimensional extent of corneal deflection without requiring external proprietary software or manual calibration.

In this study, we introduce corneal deflection volume (DeflVol), a geometric model of corneal deflection at the HC phase under the standardized air-puff loading protocol of the Corvis ST, proposed as a metric of corneal deformability. This model assumes corneal axisymmetry during air-puff-induced deformation, allowing deflection volume to be estimated from the two-dimensional Scheimpflug cross-section.

## 2 Methods

Model development and evaluation comprised three components: (i) construction of an axisymmetric two-sphere geometric model for estimating DeflVol at HC (Figure 1); (ii) evaluation against device-reported measurements not used as model inputs; and (iii) assessment of sensitivity to corneal asphericity and regular anterior toricity.

**FIGURE 1:**
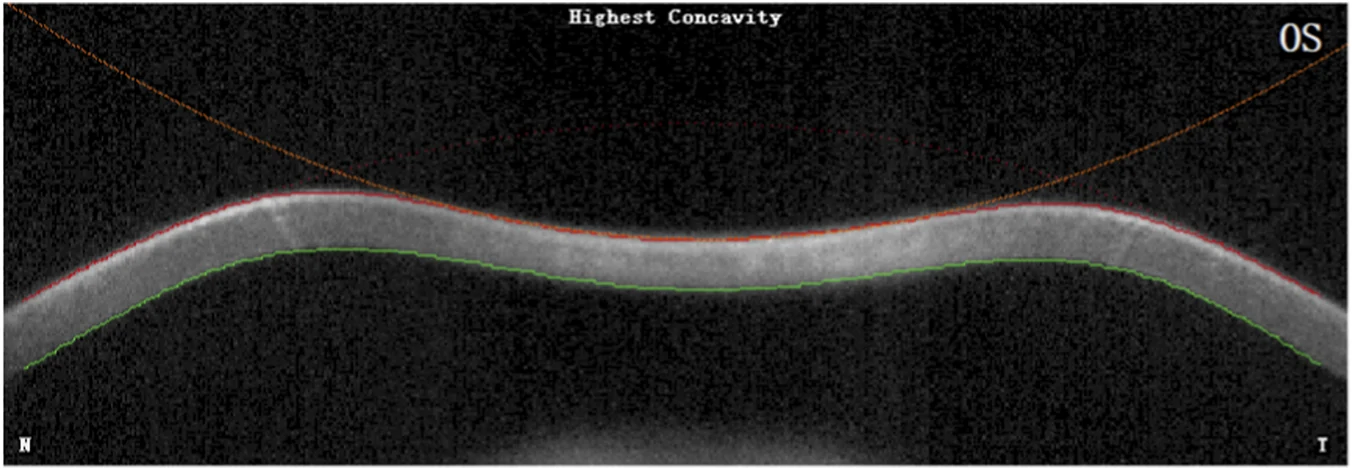
The dynamic corneal response (DCR) region at the highest concavity (HC) moment. The red solid line indicates the anterior corneal surface and the green solid line indicates the posterior corneal surface. The red dense dashed line represents the inverse curvature radius (IR) of the deflection; the red sparse dashed line represents the anterior corneal curvature radius (CR) in the initial state.

### 2.1 Geometric construction of the deflection model

At the HC moment, a cylindrical coordinate system (*ρ*, *θ*, *z*) is adopted, where the *z*-axis coincides with the corneal optical axis and is taken as positive in the anterior-to-posterior direction, and *ρ* denotes the radial distance from the *z*-axis. The corneal surface is assumed to be rotationally symmetric about the *z*-axis, so that any cross-section at axial position *z* is fully described by a single radius *ρ*(*z*). The origin is placed at the undeformed corneal apex (*z* = 0).

Two spheres are defined under this convention. The pre-deflection corneal sphere has an anterior corneal curvature radius (CR), with its center at *z*_cornea_ = CR, so that it is tangent to the plane *z* = 0 at the apex. At HC, the central cornea is concave anteriorly, and the fitted inverse-curvature sphere has an inverse curvature radius (IR), with its center anterior to the deflected apex; since the apex has been displaced posteriorly by DeflAmp, its center lies at *z*_inverse_ = DeflAmp − IR.

The axial distance between the two sphere centers is given by Equation 1:

$$d=z_{c\text{ornea}}-z_{\text{inverse}}$$ (1)

which, with the center positions above, reduces to an explicit function of the measured DeflAmp:

d=CR+IR−DeflAmp (2)

At an arbitrary axial position *z*, the radii generated by the two spheres are given by Equation 3:

$$\rho_{\text{cornea}}\left(z\right)=\sqrt{{\text{CR}}^{2}-{\left(z-z_{\text{cornea}}\right)}^{2}},\rho_{\text{inverse}}\left(z\right)=\sqrt{{\text{IR}}^{2}-{\left(z-z_{\text{inverse}}\right)}^{2}}$$ (3)

The predicted two-dimensional overlap area in the sagittal plane (predicted DeflArea) is obtained by integrating the chord width of the overlap region, which at each *z* equals twice the smaller of the two radii:

$$\text{predicted DeflArea}=\int_{z_{\min}}^{z_{\max}}2\cdot \min\;\left\{\rho_{\text{cornea}}\left(z\right),\rho_{\text{inverse}}\left(z\right)\right\}dz$$ (4)

The integration bounds are given by the intersection of the two spheres’ axial extents (Equations 5, 6):

$$z_{\min}=\max\left(z_{\text{cornea}}-\text{CR},z_{\text{inverse}}-\text{IR}\right)$$ (5)

$$z_{\max}=\min\left(z_{\text{cornea}}+\text{CR},z_{\text{inverse}}+\text{IR}\right)$$ (6)

With the center positions defined above, the overlap bounds reduce to *z*_min_ = 0 and *z*_max_ = DeflAmp, corresponding to the undeformed and deflected apices, respectively.

The two profiles *ρ*_cornea_(*z*) and *ρ*_inverse_(*z*) cross at a single axial position

$$z^{*}=z_{\text{cornea}}-\frac{d^{2}+{\text{CR}}^{2}-{\text{IR}}^{2}}{2d}$$ (7)

which defines the intersection radius

$$r^{*}=\sqrt{{\text{CR}}^{2}-{\left(z^{*}-z_{\text{cornea}}\right)}^{2}}=\sqrt{{\text{CR}}^{2}-{\left(\frac{d^{2}+{\text{CR}}^{2}-{\text{IR}}^{2}}{2d}\right)}^{2}}$$ (8)

Equations 7 and 8 give the profile-intersection position *z* ^*^ and radius *r* ^*^, respectively; the modeled overlap chord is therefore 2*r* ^*^. Because the construction depends only on the relative positions of the two spheres, placing the origin at a sphere center, as in the CorDeflVol R code, is equivalent to the apex-based convention adopted here.

The predicted deflection volume is obtained by revolving the sagittal overlap region about the *z*-axis. At each axial position, the overlap has a circular cross-sectional radius *ρ*(*z*) = min{*ρ*_cornea_(*z*), *ρ*_inverse_(*z*)}; therefore,

$$\text{DeflVol}=\pi \int_{z_{\min}}^{z_{\max}}{\left[\min\left\{\rho_{\text{cornea}}\left(z\right),\rho_{\text{inverse}}\left(z\right)\right\}\right]}^{2}dz$$ (9)

For partially overlapping spheres, |CR − IR| < *d* < CR + IR, Equation 9 has the closed form

$$\text{DeflVol}=\frac{\pi {\left(\text{CR}+\text{IR}-d\right)}^{2}\left[d^{2}+2d\left(\text{CR}+\text{IR}\right)-3{\left(\text{CR}-\text{IR}\right)}^{2}\right]}{12d}$$ (10)

Equation 10, with d from Equation 2, was used for all spherical-model analyses and was verified numerically against Equation 9.

### 2.2 Assessment against Corvis ST outputs not used as model inputs

Predicted DeflArea from Equation 4 and the modeled overlap chord 2*r* ^*^ from Equation 8 were compared with measured DeflArea and peak distance (PD), respectively, to assess correspondence in sagittal area and lateral extent with the Corvis ST outputs.

The relative area shortfall was defined as

$$\text{Relative area shortfall }\left(\%\right)=\frac{{\text{DeflAre}a}_{\text{meas}}-{\text{DeflArea}}_{\text{pred}}}{{\text{DeflArea}}_{\text{meas}}}\times 100\%$$ (11)

To investigate whether the area shortfall originated from an underestimation of the deflected region’s meridional extent, the model arc length

$$L^{*}=2\text{ IR }\arcsin\left(\frac{r^{*}}{\text{IR}}\right)$$ (12)

was compared with HC deflection length (DeflLength). The arc-length difference was defined as

$$\Delta L=\text{DeflLength}-L^{*}$$ (13)

*ΔL* was used to examine the association between the model–device length difference and the DeflArea shortfall.

The same area shortfall was then examined using a deterministic geometry-based area-matching sensitivity analysis: DeflAmp was adjusted for each eye while CR and IR were held fixed until predicted DeflArea matched measured DeflArea. DeflVol was then recomputed, and the resulting increase relative to the original model estimate was recorded as the model-implied area-matched increase.

The local elasticity of DeflVol with respect to the predicted DeflArea is

$$\frac{\text{dlnDeflVol}}{\text{dln}{\text{DeflArea}}_{\text{pred}}}=\frac{\pi r^{*}\;{\text{DeflArea}}_{\text{pred}}}{2\;\text{DeflVol}}$$ (14)

The elasticity in Equation 14, evaluated at the predicted DeflArea corresponding to the reported DeflAmp, was verified by central log–log finite differences.

### 2.3 Sensitivity to departures from geometric assumptions

Departure from sphericity. Corneal asphericity was represented using the conic sag function in Equation 15.

$$s\left(r;R,Q\right)=\frac{r^{2}}{R+\sqrt{R^{2}-\left(1+Q\right)r^{2}}}$$ (15)

The conic constant was evaluated at *Q* = 0, −0.1, −0.2, −0.25, −0.3, −0.4, and −0.5; this grid includes typical anterior corneal values of approximately −0.3 to −0.2 and extends to −0.5 as a conservative extreme. Two surface configurations were evaluated: the corneal surface alone (*Q*_c_ = *Q*, *Q*_i_ = 0) and both surfaces (*Q*_c_ = *Q*_i_ = *Q*). Changes were calculated relative to the spherical baseline as a percentage for DeflVol and an absolute difference for predicted DeflArea.

Regular anterior toricity. Regular anterior toricity was represented by the meridional radius in Equation 16, using the measured flat and steep anterior radii.

$$R\left(\varphi \right)=\frac{1}{\cos^{2}\varphi /R_{f}+\sin^{2}\varphi /R_{s}}$$ (16)

DeflVol was calculated at 72 equally spaced meridional orientations over 0 ≤ *φ* < π, and the resulting estimates were averaged. The averaged estimate was unchanged at the reported precision when 48, 180, or 720 meridians were used. IR and DeflAmp were held constant across meridians because meridian-specific values were unavailable from the single horizontal Scheimpflug section provided by Corvis ST. Accordingly, this analysis quantified sensitivity to regular anterior toricity in the undeformed anterior surface under this constraint. The relative change was calculated against the spherical–axisymmetric mean-radius baseline, with CR = (*R*_f_ + *R*_s_)/2.

### 2.4 Clinical dataset

The dataset consists of paired Pentacam AXL and Corvis ST (OCULUS Optikgeräte GmbH; Wetzlar, Germany; software versions 1.26r28 and 1.7r2781, respectively) measurements collected from 1,351 young emmetropic or myopic participants at the First Affiliated Hospital of Sun Yat-sen University. Only the left eye was included to avoid statistical dependence. Key metrics, including corneal topographic and biomechanical parameters, were obtained from the preprocessed device output. Specifically, IR, DeflAmp, measured DeflArea, PD, and DeflLength at HC were obtained from the Corvis ST output. CR was defined as the mean anterior sagittal radius on the central 15° SimK ring, calculated as the arithmetic mean of the flat and steep radii reported by the Pentacam AXL (*R*_m_ = [*R*_f_ + *R*_s_]/2). The same flat and steep radii were used in the toricity analysis. Quality control was applied by filtering for reliable quality scores (QS = OK) and excluding eyes with a history of contact lens use, ocular pathology or surgery, or values outside predefined cutoffs (central corneal thickness (CCT) < 450 μm or >650 μm, CR > 9 mm, corneal astigmatism ≥6.0 D, or intraocular pressure (IOP) > 21 mmHg).

### 2.5 Statistical analysis

All analyses were performed in R (version 4.6.0; R Foundation for Statistical Computing, Vienna, Austria), with DeflVol calculated from Equation 10 using the CorDeflVol R package (version 0.1.0; https://github.com/GulumimiRen/CorDeflVol/releases/tag/v0.1.0). Predicted DeflArea from Equation 4 and the modeled overlap chord (2*r* ^*^) from Equation 8 were compared with measured DeflArea and PD, respectively, using ordinary least-squares regression. Agreement was assessed using Bland–Altman analysis, with differences defined as the model-derived minus the device-reported value; mean differences, their 95% confidence intervals, and 95% limits of agreement were calculated. Pearson correlation coefficients were calculated for the associations of DeflVol with DeflAmp and DeflArea and for the association between *ΔL* from Equation 13 and the absolute DeflArea shortfall. To assess whether the area shortfall varied with measurement magnitude, the predicted-minus-measured difference and the relative area shortfall defined in Equation 11 were separately regressed on mean DeflArea. For the geometry-based area-matching sensitivity analysis, DeflAmp was solved separately for each eye so that predicted DeflArea equaled measured DeflArea, after which DeflVol and its change from the original model estimate were recalculated. The analytic elasticity in Equation 14 was verified using central log–log finite differences. Changes produced by the asphericity and regular anterior toricity analyses were calculated relative to the spherical baseline and the spherical–axisymmetric mean-radius baseline, respectively. *p* < 0.05 was considered statistically significant.

## 3 Results

The demographic and clinical characteristics of the 1,351 individuals included in the dataset are summarized in Table 1.

**TABLE 1:** Demographic and ocular characteristics of the study population.

Characteristic	*N* = 1,351^1^
Age (years)	23.6 ± 6.4
bIOP (mmHg)	16.72 ± 1.57
CCT (μm)	546 ± 33
ACD (mm)	3.76 ± 0.26
CR (mm)	7.81 ± 0.24
IR (mm)	6.44 ± 0.66
PD (mm)	5.02 ± 0.23
DeflLength (mm)	6.45 ± 0.39
DeflAmp (mm)	0.93 ± 0.09
DeflArea (mm^2^)	3.35 ± 0.45

1 Mean ± SD.

Abbreviations: bIOP, biomechanically corrected IOP; CCT, central corneal thickness; ACD, anterior chamber depth; CR, corneal curvature radius of the anterior surface; IR, inverse curvature radius; PD, peak distance; DeflLength, Deflection Length; DeflAmp, Deflection Amplitude; DeflArea, Deflection Area.

### 3.1 Internal geometric consistency supported the spherical assumption within the DCR region

Predicted DeflArea closely tracked measured DeflArea (*R* ^2^ = 0.99, *p* < 0.001), with a regression slope of 0.96 (Figure 2A). Bland–Altman analysis revealed a bias of −0.181 mm^2^ (95% CI, −0.184 to −0.179 mm^2^) between predicted and measured DeflArea; the 95% limits of agreement ranged from −0.276 mm^2^ to −0.086 mm^2^ (Figure 2B).

**FIGURE 2:**
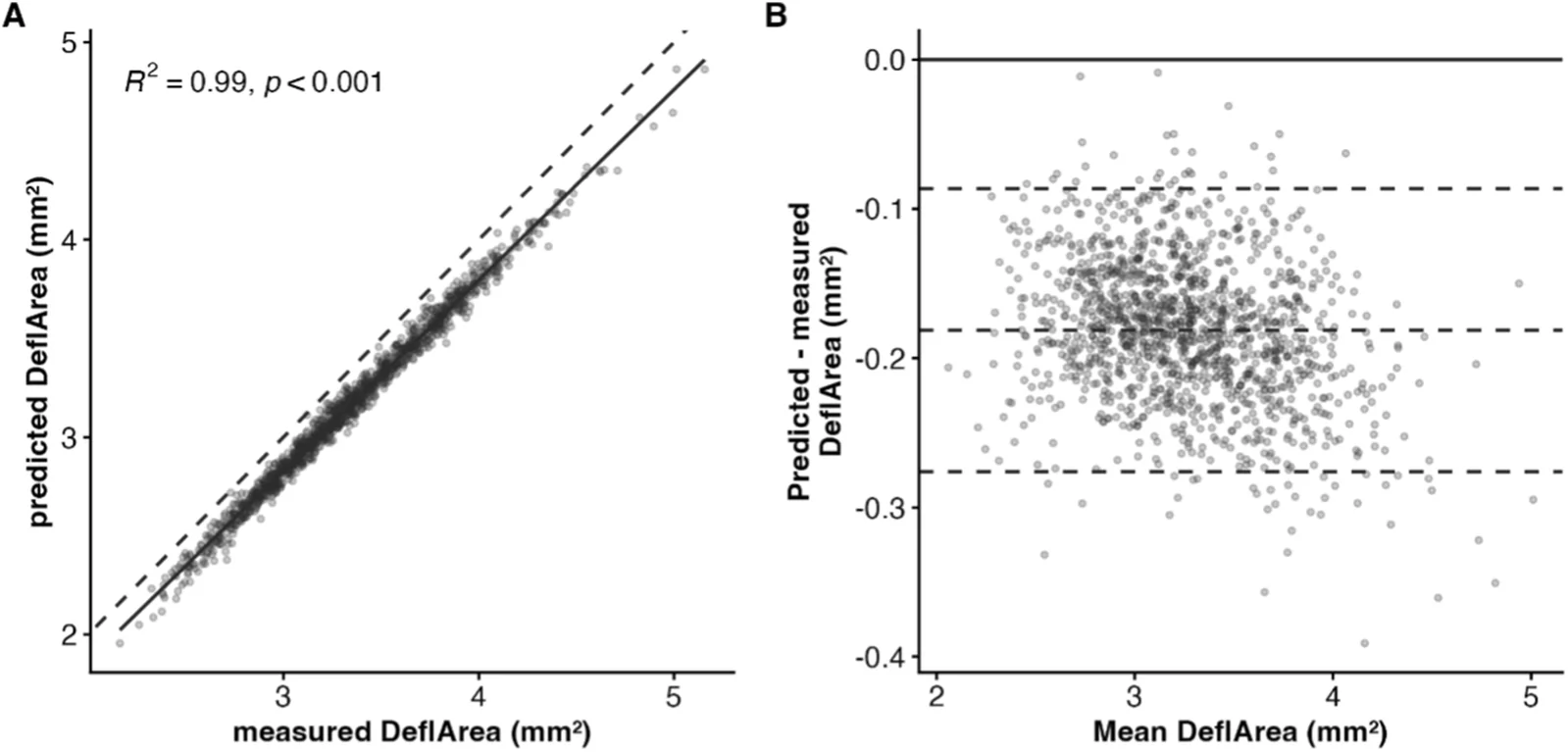
Comparison of model-predicted and measured deflection area (DeflArea). **(A)** Scatterplot of predicted *versus* measured DeflArea, with the linear regression line (solid) and the line of perfect agreement (dashed, *y* = *x*). **(B)** Bland–Altman plot of the predicted-minus-measured DeflArea difference against the mean of the two values. The middle dashed line represents the mean bias, while the upper and lower dashed lines denote the 95% limits of agreement.

Modeled 2*r* ^*^ from Equation 8 closely tracked measured PD (*R* ^2^ = 0.84, *p* < 0.001), with a regression slope of 0.95 (Figure 3A). Bland–Altman analysis revealed a bias of +0.008 mm (95% CI, +0.003 to +0.013 mm) between 2*r* ^*^ and PD; the 95% limits of agreement ranged from −0.19 mm to +0.20 mm (Figure 3B).

**FIGURE 3:**
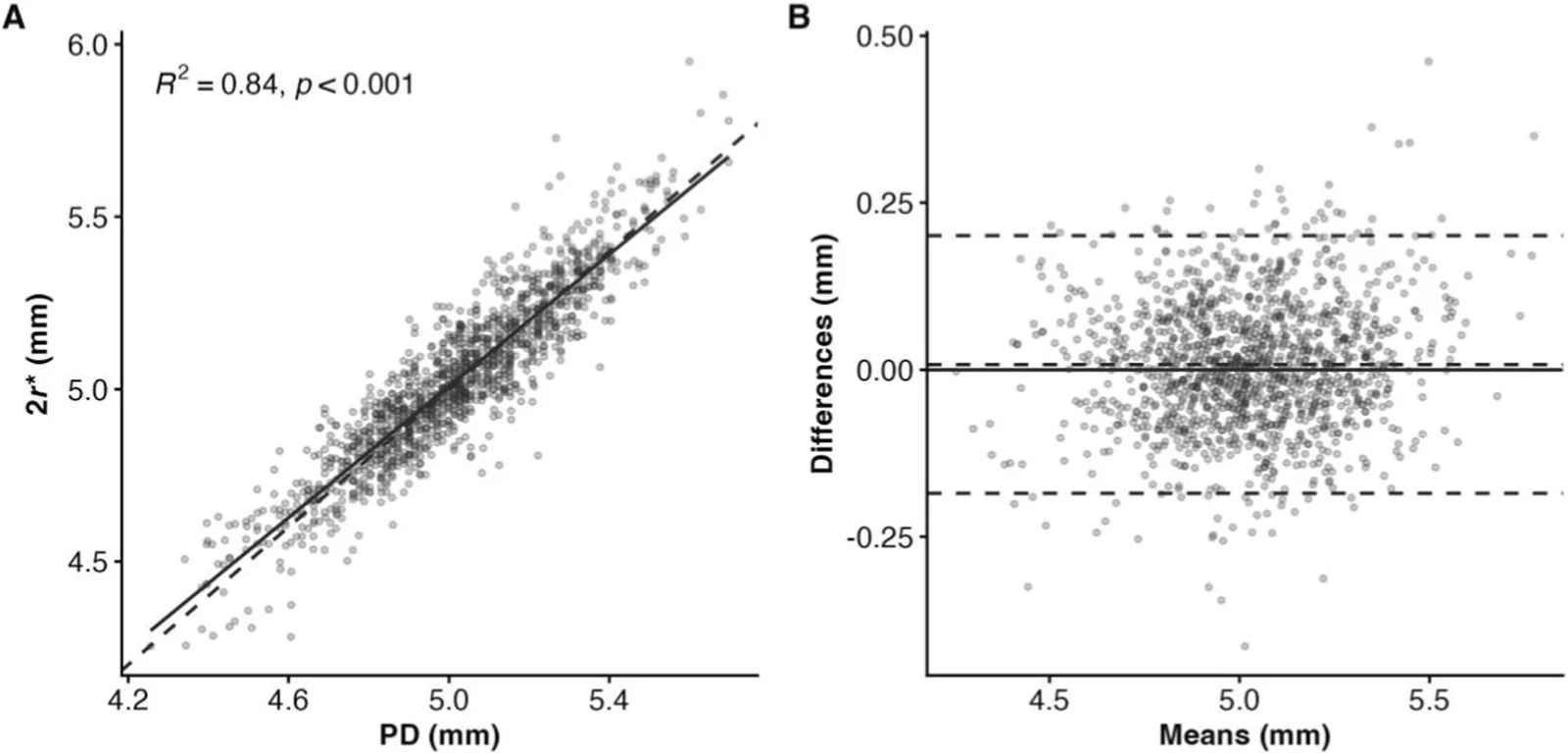
Lateral comparison of the modeled overlap chord with Corvis ST peak distance. **(A)** Scatterplot of the modeled overlap chord 2*r* ^*^—where r^*^ is the profile-intersection radius—versus peak distance (PD) at highest concavity (HC), with the linear regression line (solid) and the line of perfect agreement (dashed, *y* = *x*). **(B)** Bland–Altman plot of the difference (2*r* ^*^ − PD) against the mean of the two quantities. The middle dashed line represents the mean bias, while the upper and lower dashed lines denote the 95% limits of agreement.

### 3.2 DeflVol showed strong associations with Corvis ST deflection metrics

DeflVol was 9.48 ± 1.72 μL (mean ± SD; median, 9.34 μL; IQR, 8.26–10.61 μL). DeflVol was strongly correlated with the corresponding DeflAmp (*r* = 0.94, *p* < 0.001) and DeflArea (*r* = 0.99, *p* < 0.001) (Figure 4).

**FIGURE 4:**
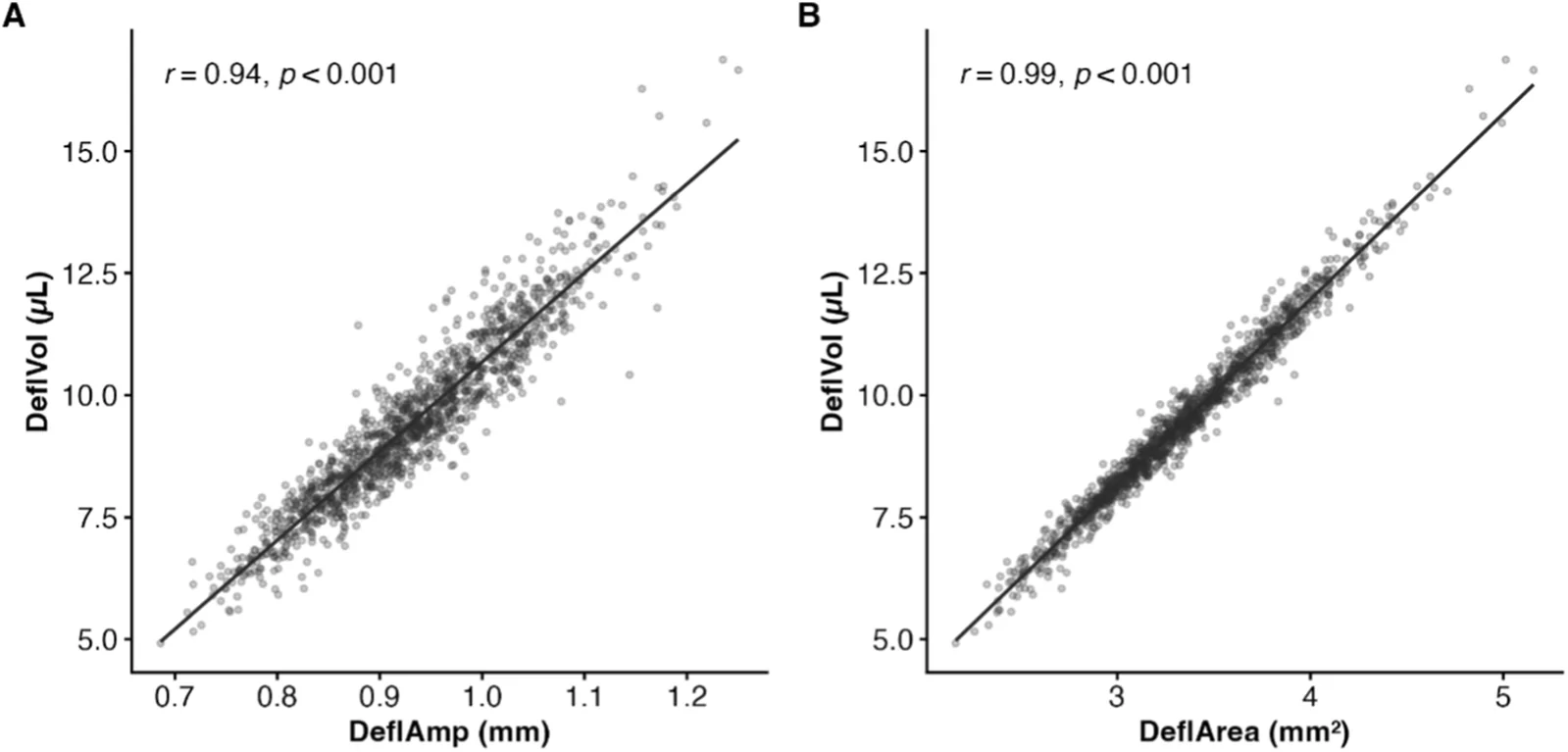
Associations of corneal deflection volume (DeflVol) with corresponding Corvis ST deflection metrics. Scatterplots of DeflVol *versus* **(A)** deflection amplitude (DeflAmp) and **(B)** deflection area (DeflArea).

### 3.3 Area shortfall is magnitude-dependent and implies a modest volumetric increase

The relative area shortfall defined in Equation 11 was 5.5% ± 1.4%. Regression of the DeflArea difference (predicted − measured) on the mean yielded a slope of −0.031 (*p* < 0.001), indicating a modest increase in the absolute shortfall with increasing mean DeflArea. Regression of the relative shortfall on the same mean yielded a slope of −0.81 percentage points per 1 mm^2^ (*p* < 0.001), indicating a modest decrease in the relative shortfall with increasing mean DeflArea. The modeled arc length *L* ^*^ from Equation 12 was 5.17 ± 0.25 mm *versus* 6.45 ± 0.39 mm for DeflLength, yielding *ΔL* = 1.28 ± 0.22 mm as defined in Equation 13; *ΔL* was correlated with the absolute area shortfall (*r* = 0.54, *p* < 0.001).

In the geometry-based area-matching sensitivity analysis, DeflAmp was adjusted until predicted DeflArea matched the measured value (mean increment, 0.036 ± 0.009 mm), after which DeflVol was recomputed. Relative to the original model estimate, the resulting model-implied DeflVol increase was 7.8% ± 2.2% (median, 7.7%; 2.5th–97.5th percentile range, 3.8%–12.2%), corresponding to approximately 0.7 μL relative to a mean DeflVol of 9.5 μL. The mean of the per-eye local elasticities defined in Equation 14 was 1.33, indicating that, within the model, a small relative change in predicted area produces approximately 1.3 times that relative change in volume (Figure 5).

**FIGURE 5:**
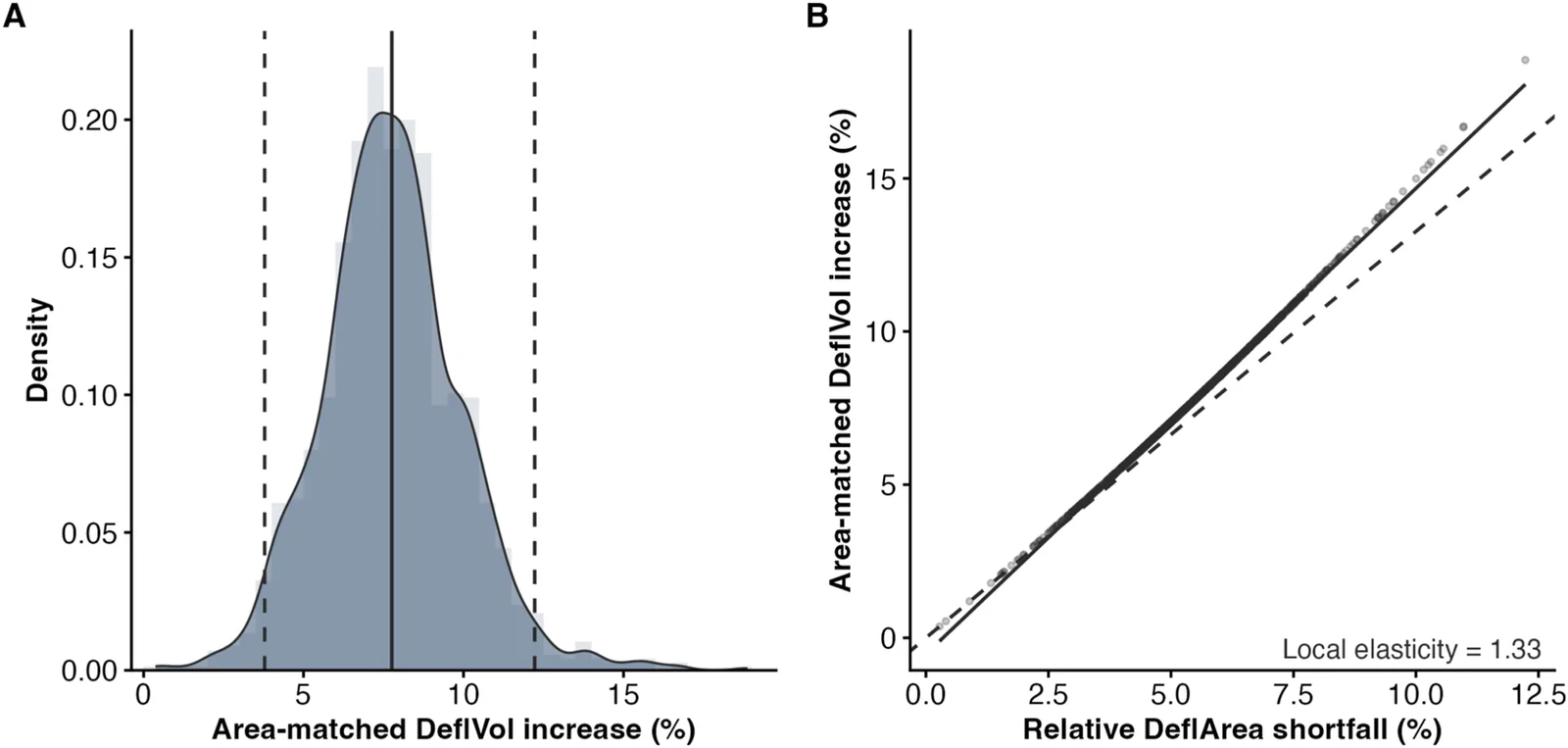
Geometry-based area-matching sensitivity analysis. For each eye, deflection amplitude (DeflAmp) was adjusted, with the curvature radii held fixed, until predicted deflection area (DeflArea) equaled measured DeflArea. **(A)** Kernel-density distribution of the resulting model-implied increase in corneal deflection volume (DeflVol) relative to the original model estimate; the solid vertical line denotes the mean, and the dashed vertical lines denote the 2.5th and 97.5th percentiles. **(B)** Model-implied DeflVol increase *versus* relative DeflArea shortfall; the solid line is the ordinary least-squares fit, and the dashed line has a slope of 1.33, equal to the mean of the per-eye local elasticities, dln (DeflVol)/dln (DeflArea_pred_).

### 3.4 Tested asphericity and regular anterior toricity produced minimal deviations in DeflVol

Departure from sphericity. Introducing asphericity on the undeformed corneal surface alone changed DeflVol, on average, by +0.16% at *Q* = −0.20 and by +0.24% at *Q* = −0.30, and applying the same *Q* to both surfaces changed it by +0.48% and +0.71%, respectively; the mean deviation remained below 1.2% even at *Q* = −0.50 (Figure 6A). At the representative value *Q* = −0.25, the predicted DeflArea increased by only approximately 0.003 mm^2^ when asphericity was applied to the corneal surface alone and by approximately 0.009 mm^2^ when it was applied to both surfaces; these increases corresponded to less than 5% of the observed area bias of −0.181 mm^2^; even at *Q* = −0.50 the increase reached only approximately 0.017 mm^2^, or about 9% of that bias.

**FIGURE 6:**
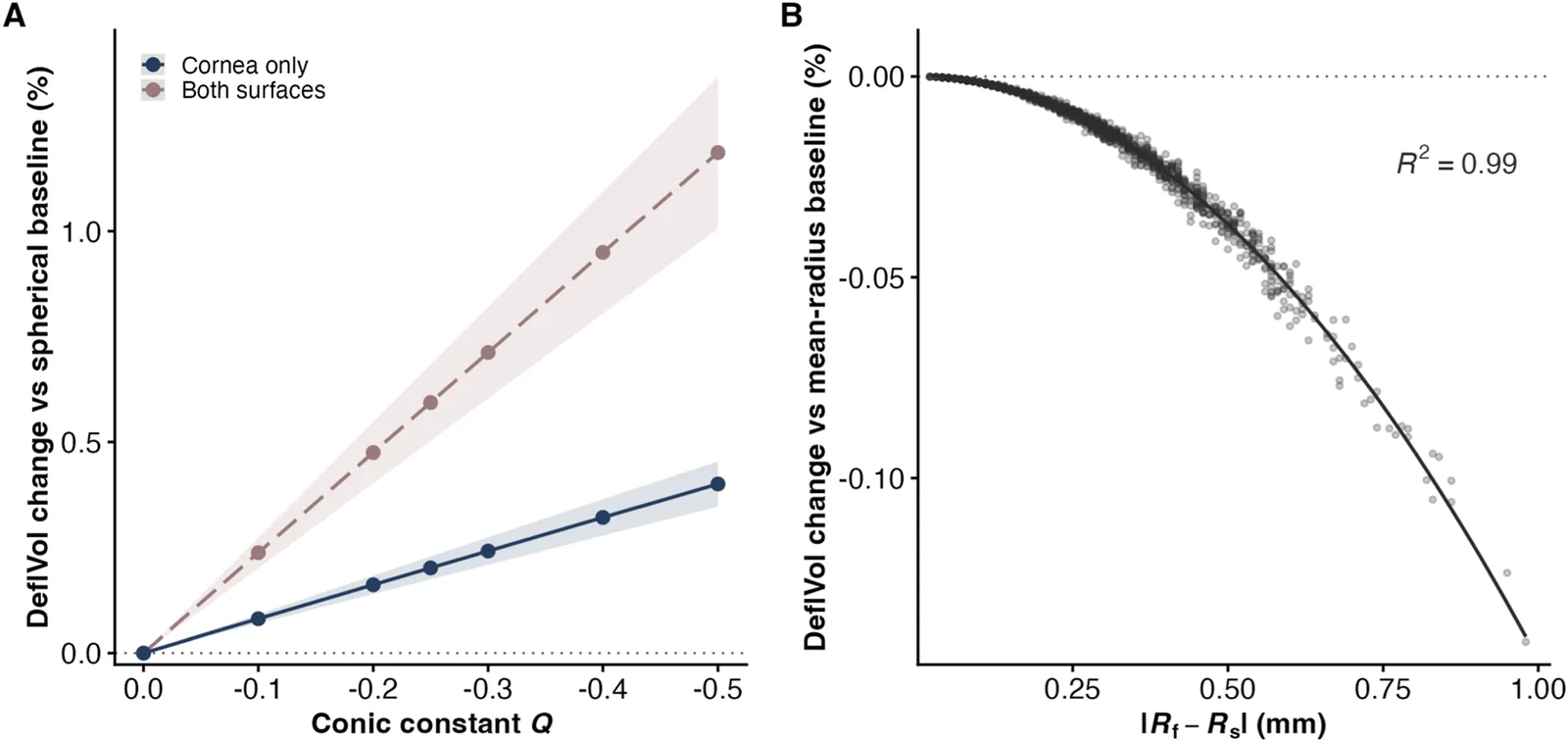
Sensitivity of corneal deflection volume (DeflVol) to asphericity and regular anterior toricity. **(A)** Mean percentage change in DeflVol relative to the spherical model. *Q*_c_ and *Q*_i_ denote the conic constants assigned to the undeformed corneal and inverse-curvature surfaces, respectively; the spherical baseline is defined by *Q*_c_ = *Q*_i_ = 0. Changes are shown as a function of the tested conic constant *Q* for asphericity applied to the corneal surface alone (*Q*_c_ = *Q*, *Q*_i_ = 0) and to both surfaces (*Q*_c_ = *Q*_i_ = *Q*); shaded bands show ±1 SD across eyes. **(B)** Per-eye percentage change in DeflVol relative to the spherical–axisymmetric mean-radius baseline, CR = (*R*_f_ + *R*_s_)/2, *versus* the absolute principal-meridian radius difference |*R*_f_ − *R*_s_|. *R*_f_ and *R*_s_ are the measured flat and steep anterior corneal radii, respectively; IR and DeflAmp were held constant across meridians. Pale points represent individual eyes, the solid curve is a quadratic fit, and the dotted horizontal line denotes zero.

Regular anterior toricity. Relative to the spherical–axisymmetric mean-radius baseline, the tested representation of regular anterior toricity, using the measured flat and steep radii while holding IR and DeflAmp constant across meridians, changed DeflVol by −0.017% ± 0.017% (99th percentile of the absolute change, 0.084%; maximum, 0.141% in the single eye with |*R*_f_ − *R*_s_| = 0.98 mm) (Figure 6B). This result quantifies the effect of regular anterior-surface toricity with IR and DeflAmp held constant across meridians.

## 4 Discussion

In this study, we developed DeflVol, a model-derived volumetric parameter based on a two-sphere model to estimate corneal deflection volume at the HC moment in Corvis ST imaging. Comparison with device outputs not used in the model derivation supported the internal geometric consistency of the spherical approximation within the DCR region. We further quantified the model–device area difference and tested the model’s sensitivity to corneal asphericity and regular anterior toricity: first, predicted DeflArea showed limited systematic underestimation relative to the Corvis ST reference values (mean bias, −0.181 mm^2^; relative shortfall, 5.5%), consistent with peripheral portions of the deflected region extending beyond the model boundary; in the area-matching sensitivity analysis, this difference corresponded to a model-implied DeflVol increase of 7.8% ± 2.2%, or approximately 0.7 μL for a mean DeflVol of 9.5 μL; second, asphericity at physiologically representative values (*Q* = −0.25 to −0.30) altered DeflVol by less than 1% on average, and regular anterior toricity produced changes of −0.017% ± 0.017% (99th percentile of the absolute change, 0.084%). Together, these results characterize the magnitude of the model–device area difference and show that the tested asphericity and regular anterior toricity had minimal effects on DeflVol among the studied eyes.

DeflArea provides a direct link between the device-derived representation and the proposed three-parameter model. Corvis ST–reported DeflArea is a two-dimensional measure of the displaced area obtained from image-derived corneal profiles in a single horizontal Scheimpflug section, whereas predicted DeflArea is calculated analytically from two smooth spherical profiles defined by CR, IR, and DeflAmp (Vinciguerra et al., 2016a; Vinciguerra et al., 2016b). The image-derived contour representation can retain local variation along the recorded meridian, including shallow peripheral deformation, while the two-sphere model uses constant-curvature profiles and bounds the modeled region at the analytic intersection *r* ^*^. This distinction is consistent with the strong linear relationship between predicted and measured DeflArea (*R* ^2^ = 0.99; slope = 0.96) together with a mean bias of −0.181 mm^2^. The near-zero bias between 2*r* ^*^ and PD indicates a small average difference between these two measures of lateral extent, whereas *L* ^*^ was shorter than DeflLength and the resulting arc-length difference was correlated with the absolute DeflArea shortfall, suggesting that part of the residual difference arises beyond the modeled boundary. Differences in initial geometry (Pentacam-derived CR *versus* the Corvis ST baseline profile), image sampling and contour processing, whole-eye-movement correction, and boundary definition may also contribute. In the geometry-based area-matching sensitivity analysis, varying DeflAmp while holding CR and IR fixed yielded a model-implied DeflVol increase of 7.8% ± 2.2% (approximately 0.7 μL), quantifying how the observed two-dimensional difference propagates to the volumetric estimate within the model.

Despite the internal geometric consistency observed for the two-sphere model in the present sample, several limitations warrant acknowledgment. First, DeflVol characterizes corneal deformation at a single time point corresponding to HC. Transient instability during corneal buckling may manifest as wobbling and high-frequency oscillations in high-speed imaging (Kling and Marcos, 2013), which may not be fully represented by a deterministic single-frame model. DeflVol therefore describes the deformation geometry at HC rather than the complete time-dependent biomechanical response. Second, the model was evaluated only in young emmetropic or myopic eyes. Ectatic and postsurgical corneas may exhibit greater geometric irregularity and biomechanical heterogeneity and may therefore show larger or less predictable departures from the model assumptions (Wang et al., 2025). Further evaluation in these and other structurally irregular corneas is therefore warranted (Wu et al., 2019; Huo et al., 2024).

In summary, DeflVol, derived from a two-sphere geometric model, provides an estimate of corneal deflection volume at HC and was strongly associated with established Corvis ST deflection parameters among the studied eyes. The area-matching analysis quantified the model-implied volumetric change corresponding to the DeflArea shortfall, whereas the asphericity and regular anterior toricity analyses produced only minimal changes in DeflVol. Together, these findings support DeflVol as a complementary, model-derived three-dimensional descriptor of corneal deformation. Future work should prioritize test–retest repeatability, evaluation in structurally irregular corneas, and comparison with volumetric estimates obtained through elevation-map integration or finite-element modeling (Joda et al., 2016; Chen et al., 2023).

## Data Availability

The raw data supporting the conclusions of this article will be made available by the authors, without undue reservation.
